# Preferred Distance in Human–Drone Interaction

**DOI:** 10.3390/vision8040059

**Published:** 2024-10-01

**Authors:** Elisabeth Maria Wögerbauer, Christoph von Castell, Robin Welsch, Heiko Hecht

**Affiliations:** 1 Department of Psychology, Johannes Gutenberg-University Mainz, 55122 Mainz, Germany; castell@uni-mainz.de (C.v.C.); hecht@uni-mainz.de (H.H.); 2 Department of Computer Science, Aalto University, 02150 Espoo, Finland; robin.welsch@aalto.fi

**Keywords:** human–drone interaction, interpersonal distance, drone appearance, personal space, proxemics

## Abstract

In two augmented-reality experiments, we transferred the paradigm of interpersonal distance regulation to human–drone interaction. In the first experiment, we used a simple spherical drone model and explored how both hovering height and approach angle affect the preferred distance. Drone height above the ground had a strong effect. The preferred distance to the drone was larger than that typically found toward human actors, in particular, when the drone trajectory was very high. In the second experiment, we sought to gain a deeper understanding of the factors that may influence this effect. In addition to the simple spherical drone model used in the first experiment, we also varied its appearance and attachment to the ground. Surprisingly, anthropomorphic features increased preferred distances. We, therefore, discuss the extent to which social aspects and subjectively perceived danger influence the preferred distance for interaction with drones, which thus need to be considered in the design of human–drone interaction.

## 1. Introduction

When asking a stranger for directions, people usually maintain a comfortable distance of about 1 m [1]. Theories of proxemics, the science of how people use space, hypothesize that this distance requirement reflects an invisible, bubble-shaped personal space boundary around a person [2]. We have shown that the shape of this space is remarkably circular in unencumbered spaces, such as in the encounter outlined above. And this preferred *interpersonal distance* (IPD) is mutual and not affected by the approach direction. In other words, we prefer roughly 1 m distance toward a person, regardless of whether she faces us, stands beside us, or is even located behind our back [1]. These results support the bubble theory of personal space, but it should be noted that it has thus far only been tested for the horizontal dimension.

In principle, the concept of personal space extends to human–robot interactions [3,4]. Studies show that humans prefer to keep a distance of around 50–100 cm from a small humanoid robot (121 cm tall) on a horizontal plane [5]. While some studies report differences in human characteristics, such as age, gender, and familiarity, or robot characteristics [4,5], meta-analytic analysis has yet to show a consistent effect pattern. Leichtmann and Nitsch [6] report a heterogeneity of methods and many inconsistencies among studies of human–robot interaction. Given the proliferation of service robots, it seems fundamental to investigate human–robot space requirements in general and the bubble theory in particular.

Some robotic systems, like drones, operate with degrees of freedom on a vertical plane, adding a third dimension to proxemics of human–robot interaction, which opens up new research questions: Do interpersonal space requirements transfer to non-human objects such as a delivery drone or a robot? Does the comfortable distance we like to maintain toward a drone follow similar rules as human IPD? We conducted two experiments to begin to answer these questions. Before reporting them, we briefly discuss relevant existing findings regarding IPD as well as some considerations of drones that we are likely to encounter.

### 1.1. IPD

The concept of a circular personal space can be traced back to considerations entertained by Gestalt psychologists about 100 years ago, in particular by Kurt Lewin [7] and William Stern [8], and it was popularized in the 1960s by Edward Hall [9] and Heini Hediger [10] before regaining recent attention. Personal space has been thought of as a three-dimensional space that behaves like a soap bubble [11]. When no forces act on it, it assumes the shape of a sphere, but as soon as forces do act on it, it transforms into other shapes, for instance, into an ellipsoid. Although time-honored, the bubble hypothesis has not yet been rigorously tested. Such a test might have been considered dispensable because there were no everyday occasions where a person would approach us from above or below, beyond the ground plane. However, this may be the normal use case for a future delivery drone. When considering the vertical dimension, the shape of the bubble, and thereby IPD, should be affected by the height of the drone above the ground or by the ceiling height in enclosed spaces. The only study that has considered bubble deformation in the third dimension is the one in which the authors have investigated the effect of ceiling height on preferred IPD [12]. They used a small empty room in which they could alter the ceiling height between about 2 and 3 m by means of an artificial ceiling. The subject stood at the farthest point from the door, through which an experimenter then entered and approached until the subject would say ‘stop’. The preferred IPD was 1 m for a group of subjects tested with the low ceiling but only 0.7 m for a group tested with the high ceiling. This lets the authors conclude that personal space does behave like a bubble, which is compressed when lowering the ceiling so that the bubble flattens and has to expand sideways. However, it remains questionable if the bubble model can entirely explain the effect. When we calculate the change in diameter of an air balloon that is squished from a spherical shape into an ellipsoid, it should merely increase by 11%, which would correspond to 0.78 m as opposed to the 1.0 m observed by the authors.

In our own lab, we have extensively explored situations where we have replaced the real human observers with large puppets or mannequins, with three-dimensional renditions of humans, mere pictures, and even silhouettes of people. Rather surprisingly, when the cover story of meeting a stranger in an unencumbered space is maintained, all renditions produce more or less the same preferred IPD of about 1 m (see [1]). This robustness of IPD perception across modalities is convenient and facilitates data collection. Notwithstanding this robustness, however, there are large inter-cultural and situational differences regarding preferred IPD. For instance, a very large paper-and-pencil study involving 42 countries found that the preferred IPD for a similar social situation ranges between 0.8 m and 1.2 m [13].

Interest in IPD regulation in combination with virtual-reality technology has produced numerous studies that qualify the situational factors that influence IPD preferences. Among these are emotional expression [14] and body height [15]. Not surprisingly, larger IPD is maintained in the face of angry expressions or toward a taller person. Given these and other factors that affect IPD, which are too numerous to mention here, it is likely that perceivable attributes of non-human objects, such as robots or drones, likewise modify the distance we would like to maintain toward them.

### 1.2. Drones

Recent technological advances, among others, in electrical energy storage, such as lithium-ion batteries, have made powerful drones affordable. Given the many potential uses, for instance, of delivery drones, which can deliver packages much faster than ground-based services, it appears safe to predict that drones of manifold shape and purpose will become commonplace and that encounters with drones will become ubiquitous. It is, therefore, important to know the conditions that drive human distance requirements and preferences during encounters with drones. Research on this topic has begun from an engineering perspective (see, e.g., [16]) but is still scarce in the domain of human factors psychology. One notable exception is a study by Duncan and Murphy [17] who conducted a classical IPD study with a small drone. They suspended a quad-rotor helicopter with a diameter of 1 m from a track on the ceiling of a standard classroom. The helicopter drone could be either 1.5 m or 2.1 m above the ground. Their subjects were on average 1.7 m tall and had to stand upright in a fixed position. As the drone approached and passed the subject on a diagonal trajectory with a maximal proximity of 0.6 m, the subject had to indicate at which point the drone had reached and was exceeding a comfortable passing distance. This distance ranged between 0.6 m and 0.9 m as measured to the subjects’ outer edge, presumably along one leg. When extrapolating to the distance toward the body center, this is roughly compatible with the preferred IPD between two people in an open space, which we found to be about 1 m as measured from the vertical body axes. Interestingly, in Duncan and Murphy’s study [17], drone height above the ground had no effect on the closest comfortable approach distance. A possible reason for this finding could be that the experimental setup, with the drone mounted on a moving platform and the briefing on minimal safety risks, provided a high level of safety to the subjects, which likely influenced the dependent variables.

### 1.3. The Bubble Hypothesis Applied to Drones

However, when taking the bubble metaphor seriously as a theoretical basis, drone height should alter the preferred IPD. Maybe the authors had not varied drone height significantly. Compared to the reference case of a drone that enters our personal space flying at a height corresponding to the center of our bubble, we should be willing to tolerate closer passage trajectories if the drone is flying higher, that is, above us. Depending on where exactly we locate the center of our bubble, the effect would vary in magnitude. If this center is at chest height, raising the drone from chest height to 2.1 m should produce a noticeable effect. However, if the bubble center is at eye level, raising the drone to 2.1 m might not make enough of a difference to be measurable with a standard IPD paradigm. Thus, predictions are dependent on the exact shape of the bubble as well as on its exact origin. The bubble may be flattened at the top (and bottom) because our interactions, throughout evolution, have been mostly confined to the ground plane. It may also vary as a function of our body proportions. For now, we avoided the case where there might be a singularity in the bubble; that is, the situation where the drone is directly above our head, and might pose an increased risk of dropping on us, will not be considered here. If the personal space in human–drone interactions is approximately spherical, we should be willing to tolerate closer distances toward an approaching drone when it is higher above the ground, as opposed to lower positions in front of the observer.

As a first attempt to map out the space between a drone and a human observer (Experiment 1), we have decided to systematically vary the height above the ground of the drone’s approach trajectory and to use a stop distance paradigm in which the human observer is able to remotely move the drone back and forth on each of these given linear trajectories until the closest comfortable distance has been reached. This is an extension of previous research, both in terms of investigating a much wider range of drone heights and in terms of aligning drone height with eye level to reflect personal space. We were also concerned with perceptual compression effects that may arise when there is uncertainty about how the drone position relates to the ground surface (see [18]). Thus, in Experiment 2, we added a condition where the drones had feet as if sliding across the ground plane. Finally, we varied the facial expression by adding sketched eyes and ears and thus presenting an indicator of anthropomorphism. We use Augmented Reality (AR) to vary the depiction of the drone systematically while also maintaining external validity [3,6]. This provides insight into how specific possible features of a drone may affect the comfortable interaction distance.

## 2. Experiment 1

To gain insights into the basic shape of the bubble, we varied both the height of the drone and the angle at which it approached the subjects in this experiment.

### 2.1. Subjects

Twenty-eight subjects (thirteen female, fourteen male, and one non-binary) with an average age of 27.79 years (*SD* = 9.23 years) completed the study. They received partial course credit for their participation. The study was conducted in line with the ethical standards of the Local Ethics Board of the Department of Psychology of Mainz University. Since voluntary participation on a fully informed basis and anonymity were assured, and there was no risk of physical stress or disadvantages due to group assignment, the ethics board deemed the approval of this experiment unnecessary. All subjects were informed about the voluntary nature of their participation and provided written consent. All subjects had normal or corrected-to-normal vision.

Most of the subjects had no direct experience with drones (15 subjects). Among the remaining subjects, the majority had been present during drone flights primarily for recording purposes, while a few had encountered drones as toys. Only three subjects had previously controlled a drone themselves. Regarding AR, 20 out of the 28 subjects had no prior experience, while the rest had interacted with AR in the context of other experiments, a museum visit, or a smartphone game.

### 2.2. Apparatus and Drone Appearance

**Figure 1:**
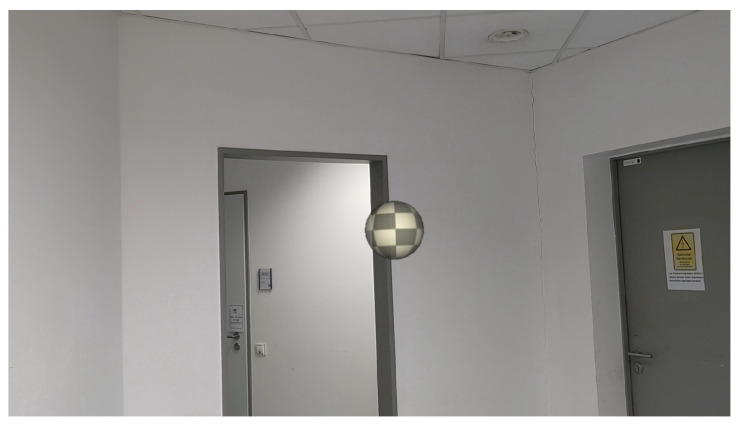
Depiction of the projected drone in the room as seen through the HoloLens 2.

An AR headset (HoloLens 2) with a visible field of view of approximately 29° vertical and 43° horizontal and with optical see-through capability was used for the experiment. Subjects could see the real environment through the AR headset, with an augmented spherical object referred to as a communication drone (25 cm diameter, white-gray checkerboard texture) projected into the room (see Figure 1). The anchor point for the drone was at the center of the sphere, and all height measurements refer to this central point. The drone emitted a humming sound, which varied in volume based on the distance to the subject and was played through the AR headset. This simple representation was chosen to minimize the influence of technical aspects such as moving rotors, sharp edges, and unpleasant noises.

**Figure 2:**
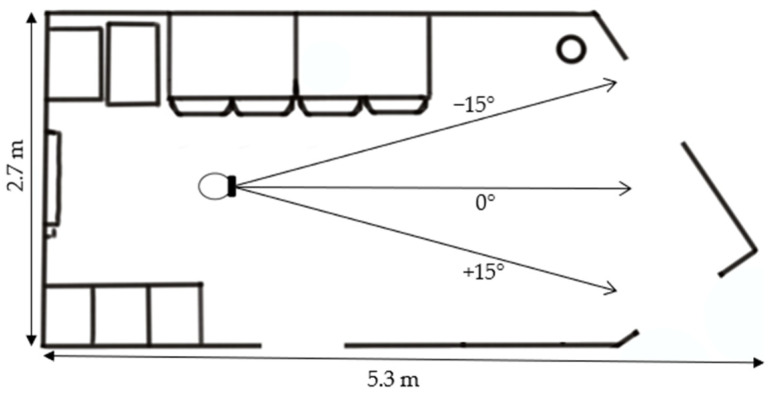
Experimental setup in Experiment 1. The subject stood at the marked point in the room, facing in the designated direction. The ceiling height was 3 m. The drone could be displaced along the respective marked trajectories and kept the same hovering height throughout a trial. The room was a common room/meeting area furnished with a small tea kitchen, tables, and chairs.

Subjects were instructed that the sphere was a drone named ALACS (‘Airborne Local Autonomous Communication System’) and that its function was to interact verbally with humans. We chose this framing to increase comparability with studies on interpersonal distance (e.g., [1]) and to make the indoor use of a drone plausible. The experimental setup is depicted in Figure 2. The simulation was created with Unity Version 2021.3.6f1 and transmitted wirelessly to the AR headset (using the Holographic Remoting function).

### 2.3. Design and Task

The design was a two-factorial within-subjects design with the height of the drone and angle of approach to the subject as independent variables. The height was presented at eight different levels (10–150% of the subject’s eye level, in steps of 20%), and the angle had three levels (−15°, 0°, +15°). This resulted in 24 different combinations of height and angle, each presented six times with different starting distances (ranging from 2.2 to 3.2 m, in increments of 0.2 m) to avoid anchoring effects. Thus, each subject completed a total of 144 trials, presented in random order. There were no practice trials. The subjects’ task was to adjust a comfortable distance for communication with the drone using a mouse wheel. This comfortable interaction distance was calculated as the horizontal distance between the subject’s assumed central point of the head and the center of the drone. We added a fixed value for half the average sagittal head diameter to the coordinates of the head camera attached to the front of the AR headset for the assumed central point of the subject’s head.

### 2.4. Procedure

**Figure 3:**
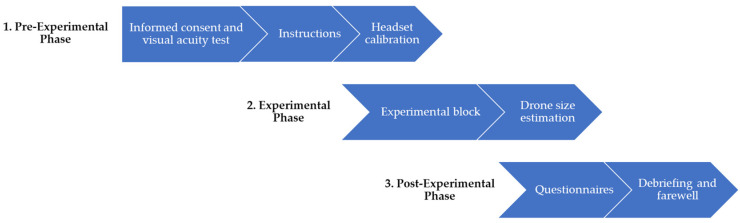
Procedure of the laboratory experiment.

The procedure is depicted in Figure 3. It began with obtaining informed consent, followed by an assessment of the subject’s visual acuity and instructions on the experiment. Next, the subject’s eye level was measured and entered into the experimental program, and the HoloLens 2 was calibrated to their eyes using the internal calibration routine.

Then, the 144 trials were presented in random order. The subjects adjusted the desired distance to the drone using the mouse wheel and confirmed it to the experimenter, who then started the next trial. They could move the drone backward and forward until satisfied with its position. The subjects were allowed to take as much time as they needed to adjust the distance and could take a break at any time. After completing all trials, the subjects indicated the size of the drone by showing the diameter of the sphere between their fingers, which was measured by the experimenter. Finally, we collected demographic data and information on technology affinity using the Affinity for Technology Interaction Scale (ATI; [19]) and the German Technology for Usage Inventory (TUI; [20]). Subjects also completed the 10-Item Big Five Inventory (BFI-10; [21]) and answered questions about their experience with drones and their attitudes toward the use of drones for various purposes.

### 2.5. Results

For each combination of subject, height, and angle, we excluded outlier data points based on a Tukey criterion of 3 interquartile ranges below the first or above the third quartile. This exclusion affected 1.46% (*n* = 59 data points) of the $n_{total}$ = 4032 data points. Next, we aggregated the data per experimental condition.

To analyze the effects of the height of the drone (with eight levels) and its angle of approach (with three levels), we conducted a repeated-measures ANOVA (rmANOVA) based on the mean comfortable interaction distance, with height and angle as independent variables. All statistical analyses were performed using RStudio 2023.12.1 and interpreted at a significance level of *α* = .05. Where indicated, we conducted pairwise *t*-tests with Bonferroni correction for follow-up comparisons.

**Figure 4:**
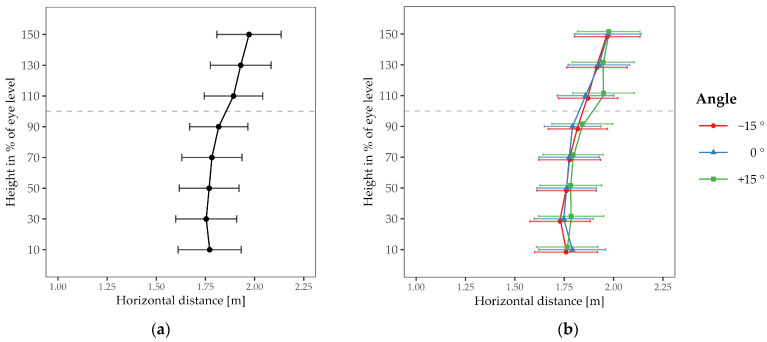
Mean comfortable distance as a function of drone height. The grey dashed line represents the subjects’ eye level. Symbols differing in shape and color represent the approach angles. Error bars indicate ± 1 SE of the mean. The left panel (**a**) shows the averaged results of the three approach angles depicted in the right panel (**b**).

The comfortable interaction distance showed a dependence on drone height (see Figure 4). When the drone was above eye level, the mean comfortable distance was 1.93 m (*SD* = 0.82 m), compared to 1.78 m (*SD* = 0.81 m) for positions below eye level. This was confirmed by the rmANOVA, which showed a significant main effect for height; *F*(1, 27) = 9.88, *p* = .004, *η*^2^*_p_* = .27. Post-hoc tests revealed that the comfortable interaction distance at 110% of the subject’s eye level (i.e., directly above the head, *M* = 1.89 m, *SD* = 0.79 m) significantly deviated from drone heights at 70% (*M* = 1.78 m, *SD* = 0.81 m; *t*(27) = 3.80, $p_{bonf}$ = .021, $d_{z}$ = 0.72), and 90% (*M* = 1.82 m, *SD* = 0.78 m; *t*(27) = 3.92, $p_{bonf}$ = .015, $d_{z}$ = 0.74), of the subject’s eye level (i.e., at chest height and directly below the head). We found no significant differences for the main effect of approach angle or for the interaction effect of height and angle (both *p* > .05).

Interestingly, the grand mean of the comfortable interaction distances to the drone (averaged over all heights and angles: 1.84 m) was considerably larger than the typical distance of about 1 m reported in studies for interactions between humans. We tested the grand mean against 1 m and found it to be significantly larger; *t*(27) = 5.60, *p* < .001, *d* = 1.06.

We calculated Pearson product–moment correlations to examine the relationship between the average comfortable interaction distance and the variables related to personality, technology affinity, and usage collected in the questionnaires. There were no significant correlations with these variables, except for a positive correlation of the mean comfortable interaction distance with the personality dimension Conscientiousness; *r* = .40, *p* = .033).

After completing the trials, subjects were asked to estimate the diameter of the spherical drone. They had a fairly accurate sense of its size; the average estimate, based on data from 19 of the 20 subjects (as this measurement was initiated from the second subject onward), was 27.96 cm (*SD* = 6.50 cm), which is very close to the actual diameter of 25 cm. They also reported that they had perceived the drone as a three-dimensional object. We assessed immersion using a subscale of the TUI questionnaire. The four items on this subscale asked subjects to rate their agreement with statements on a 7-point scale (from 1 = strongly disagree to 7 = strongly agree). The mean score for immersion was 3.79 (*SD* = 1.13), with a Cronbach’s alpha of .61.

In sum, in this experiment, we have varied the height and angle at which the drone approached the subject. We aimed to understand how the comfortable interaction distance changes based on these factors. The approach trajectories above eye level produced the largest comfort distance. The approach angle was irrelevant. Note, however, that the range of approach angles was limited to ±15 degrees due to the restricted field of view of the AR headset.

## 3. Experiment 2

To further investigate how specific visual properties of the drone influence the interaction bubble, we conducted Experiment 2, in which we presented different drone appearances. We altered the appearance of the drone to gather more information on how specific properties affect the comfort distance. In principle, one might expect anthropomorphic properties and even more basic image characteristics, such as lightness or contrast, to exert an influence [22,23,24]. We have decided to first focus on anthropomorphic aspects. We gave the drone a face and, in another condition, additional feet, and as a reference we included the original faceless drone without ground contact.

### 3.1. Subjects

Twenty subjects (sixteen female, four male, and zero non-binary) with an average age of 23.60 years (*SD* = 3.57 years) completed the study. They were instructed and debriefed exactly as in Experiment 1. They received partial course credit for their participation. The study was conducted in line with the ethical standards of the Local Ethics Board of the Department of Psychology of Mainz University. Since voluntary participation on a fully informed basis and anonymity were assured, and there was no risk of physical stress or disadvantages due to group assignment, the ethics board deemed the approval of this experiment unnecessary. All subjects were informed about the voluntary nature of their participation and provided written consent. All subjects had normal or corrected-to-normal vision.

Most of the subjects had no direct experience with drones (13 subjects). The remaining subjects had either observed drone flights primarily for recording purposes or had interacted with toy drones. Six of the subjects had previously operated a drone themselves. Fourteen of the twenty subjects had no experience with AR prior to the experiment; the rest had encountered AR in the context of other experiments, a museum visit, or a computer game.

### 3.2. Apparatus and Drone Models

**Figure 5:**
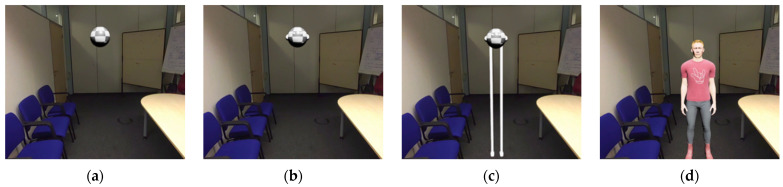
The projected drone models and the human male model in the room as seen through the HTC XR Elite: (**a**) Simple spherical form; (**b**) Variant with anthropomorphic features; (**c**) Variant with anthropomorphic features plus ground anchoring; (**d**) Human male model. All models are positioned with their anchor point at a height of 110% of eye level and at a distance of 2 m.

Due to the limited field of view of the HoloLens 2 (in particular, the feet would have been truncated because of the small vertical range), we used an XR headset (HTC XR Elite) with a rendered field of view of approximately 91° vertical and 102° horizontal, and video pass-through. The basic design of the drone was similar to Experiment 1, featuring a spherical object with a white-gray checkerboard pattern and a diameter of 25 cm. We added two variants, one with slightly anthropomorphic features (sketched eyes and ears) and one with additional feet for ground anchoring (see Figure 5). All three drone models emitted the same humming sound as used in Experiment 1, with volume modulation as a function of distance to the subject. A human male model was also included for comparison (see Figure 5). The anchor point for the drones was at the center of the sphere, and height/distance measurements refer to this central point. For the human model, height measurements refer to the top of the head.

**Figure 6:**
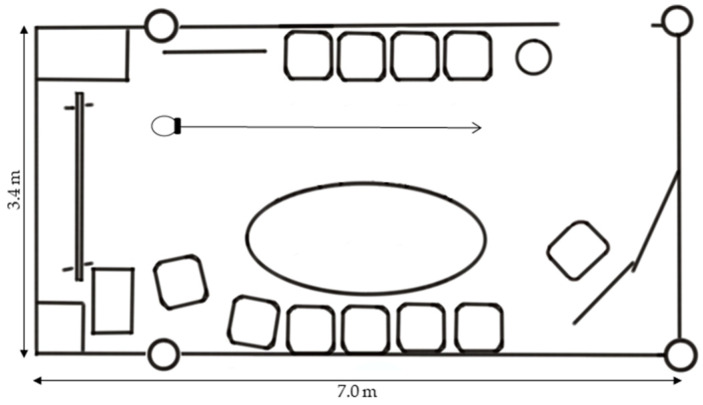
Experimental setup in Experiment 2. The subject stood at the marked location in the room, facing in the indicated direction. The room was a meeting room furnished with a table, chairs and writing surfaces.

The cover story of the drone as a communication system remained unchanged. This experiment was conducted in a different room than in Experiment 1 (see Figure 6).

The simulation was created with Unreal Editor Version 5.3.2 and transmitted to the XR headset via the Virtual Reality Preview option in Unreal.

### 3.3. Design and Task

The design was a two-factorial within-subjects design with the height of the drone and the drone model as independent variables. The approach angle was always straight ahead. The height was again varied such that the drone was presented at eight levels (10–150% of the subject’s eye level, in steps of 20%). The drone model had three levels (simple spherical form, slightly anthropomorphic features, anthropomorphic features plus ground anchoring, as shown in Figure 5). This resulted in 24 combinations of height and model, each presented six times with different starting distances (2.2 to 3.2 m, in increments of 0.2 m) to avoid anchoring effects. Each subject completed the 144 trials in separate blocks for each drone model. Within a given block, the order of trials was randomized. The sequence of blocks was counterbalanced across subjects to control for order effects. Afterward, for comparison purposes, a human male model was presented with a body height of 90% and 110% of the subject’s eye level with the same starting distances and in random order, amounting to an additional 12 trials. There were no practice trials. Subjects adjusted the drone (or human avatar) to a comfortable distance for communication using the controller thumb-stick. Again, to obtain the horizontal distance from the subjects’ assumed head center to the drone center, we added a fixed value for half the average sagittal head diameter to the coordinates of the head camera, which was embedded at the front of the XR headset.

### 3.4. Procedure

The procedure was identical to that of Experiment 1. As before, we asked for an estimate of drone diameter, demographic data, and questionnaires on technology affinity and usage, drone experience, and attitudes. Subjects were asked to rate the perceived humanness of each presented model on a 10-point scale (ranging from 1 = not at all to 10 = very much). Subjects gave a rating about the perceived realism of the objects in the room on a 10-point scale (ranging from 1 = not at all to 10 = very much), with an average rating of 7.70 (*SD* = 1.87). We also assessed immersion using the corresponding subscale of the TUI questionnaire, which resulted in a mean immersion score of 3.61 (*SD* = 1.03) on a 7-point scale, with a Cronbach’s alpha of .51.

### 3.5. Results

The data preparation and analysis followed the same procedure as in Experiment 1. For each combination of subject, height, and drone model, we excluded outliers based on a Tukey criterion of three interquartile ranges below the first quartile or above the third quartile. This resulted in the exclusion of 1.83% (*n* = 57 data points) of the $n_{total}$ = 3120 data points. The remaining data were aggregated for each experimental condition. Due to a technical error, one subject was presented with the human model at an incorrect size, so the data regarding the comfortable interaction distance to the human model include only 19 subjects.

With regard to perceived humanness, the human model received the highest rating with a mean value of 8.50 (*SD* = 1.70), followed by the variant with anthropomorphic features plus ground anchoring (*M* = 5.25, *SD* = 2.59). The variant with slightly anthropomorphic features (*M* = 4.20, *SD* = 2.33) and the basic spherical form variant (*M* = 1.90, *SD* = 1.68) were perceived as less human-like.

We conducted a rmANOVA to analyze the effect of drone height (with eight levels) and drone model (with three levels) based on the mean comfortable interaction distance. The human model is not considered in the rmANOVA since it was only presented at 90% and 110% of eye level.

**Figure 7:**
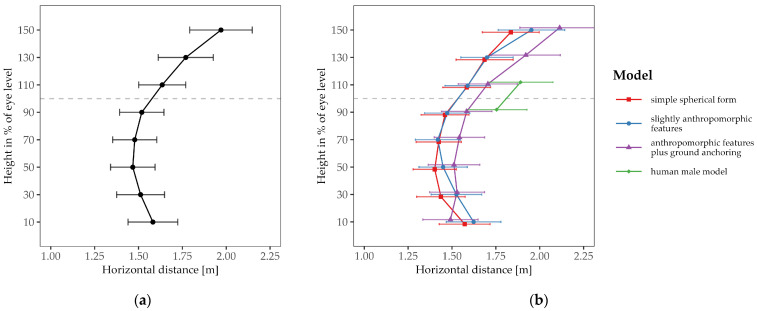
Mean comfortable distance as a function of drone height. The grey dashed line represents the subjects’ eye level. Symbols differing in shape and color represent the different models. Error bars indicate ± 1 SE of the mean. The left panel (**a**) shows the averaged results of the three variants of the drone model depicted in the right panel (**b**).

Figure 7 shows that the comfortable interaction distance was influenced by both the drone model and height. The mean comfortable distance was 1.60 m (*SD* = 0.59 m) for the simple spherical form, 1.56 m (*SD* = 0.57 m) for the variant with slightly anthropomorphic features, and 1.68 m (*SD* = 0.68 m) for the variant with additional ground anchoring. This effect of the drone model was confirmed by the rmANOVA, which showed a significant main effect of the drone model; *F*(2, 38) = 3.99, *p* = .027, *η*^2^*_p_* = .17. Post-hoc tests revealed that only the spherical form and the variant with anthropomorphic features plus ground anchoring differed significantly; *t*(19) = 2.72, $p_{bonf}$ = .041, $d_{z}$ = 0.61.

Across all three drone models, the comfortable interaction distance was greater at heights above eye level compared to below eye level (see Figure 7). The rmANOVA confirmed a significant main effect of height; *F*(1, 19) = 13.77, *p* = .001, *η*^2^*_p_* = .42. The significant interaction effect between variant and height (*F*(2, 38) = 7.53, *p* = .002, *η*^2^*_p_* = .28) indicates that the comfortable interaction distance does not simply shift the baseline between drone models; rather, subjects perceived the variants differently at various heights. The simple spherical form and the variant with anthropomorphic features showed similar comfortable interaction distances at heights between 70% and 130% of eye level. However, at extreme heights, subjects wanted a greater distance to the variant with anthropomorphic features. Some of the subjects reported that their intention was to maintain eye-contact to the drone model that had anthropomorphic features. The subjects generally preferred a greater distance to the variant with anthropomorphic features plus ground anchoring than to the other models without ground anchoring, except at the lowest height, where it was permitted to come within closest proximity to the subject. For the heights above eye level, some subjects reported the ground anchoring to be unnatural and/or unpleasant.

The grand mean of the comfortable interaction distances to the drone (averaged over all heights and drone models: 1.62 m) was significantly larger than the typical comfortable interaction distance of about 1 m reported in studies for interactions between humans; *t*(19) = 4.56, *p* < .001, *d* = 1.02.

After the experimental trials with the three drone models, a human male model was presented such that the model’s top of the head was at 90% and 110% of the respective subject’s eye level (slightly below and above eye level). Surprisingly, subjects preferred an even larger distance to the human model (*M* = 1.82 m, averaged over both heights).

As in Experiment 1, subjects indicated the perceived diameter of the drone after the experiment. The actual diameter was 25 cm, while the mean perceived diameter was 36.52 cm (*SD* = 7.25 cm), indicating an overestimation.

Finally, we calculated Pearson product–moment correlations to examine the relationship between the average comfortable interaction distance across all three drone models and the variables related to personality, technology affinity, and technology usage collected in the questionnaire. There were no significant correlations with these variables, except for a positive correlation of mean comfortable interaction distance with the personality dimension Anxiety about Technology; *r* = .48, *p* = .030.

## 4. Discussion

The objective of this study was to investigate the preferred distance in human–drone interaction and to identify the factors influencing it. The results of the two experiments showed that the comfortable interaction distance is significantly affected by the drone’s height relative to the subject’s eye level. Subjects allow drones to approach closest at about 50% of their eye level. This would correspond to a relaxed grasping position. The appearance of the drone also affected the comfortable interaction distance. Subjects preferred larger distances when the drones had anthropomorphic features.

When comparing the comfortable distances of the simple spherical form found in both experiments, a key similarity emerged (despite the different shapes of the preferred distance). The preferred distance was larger above eye level than below eye level. This may either be the result of a protective strategy toward potentially dangerous situations (an object that is located above the head is harder to fend off) or because an upward head movement results in physical discomfort and is therefore avoided. In our experiments, wearing the headsets probably intensified this effect even further. This is indicated by individual statements from subjects in Experiment 1, who felt that the extremely low and high positions of the drone were physically demanding to look at. Due to the small field of view, more and more extensive head movements were necessary for the subjects to keep the augmented object in sight when it approached particularly high or low. In Experiment 2, with the larger field of view, subjects did not express any issues with regard to the weight of the headset or when making head movements.

### 4.1. Updating Proxemic Theory for and with Human–Drone Interaction

Our results have significant implications for applying proxemic theory to human–drone interaction. When interacting with drones, personal space exhibits a distinct vertical component. Comfort distance varies with eye level in a C-shaped manner, with larger distances being preferred for approaches above eye level and at the lower leg level. This indicates a convex relationship between comfort distance and drone height rather than the concave relationship suggested by the bubble metaphor. Thus, the bubble metaphor does not hold here. Instead, an action-based deformation of personal space seems more likely. At positions where manual control or manual protection is more imminent, a larger safety distance seems to be preferred. This would be in line with studies on avatar height, which found that increasing avatar height enlarged personal space [25]. However, these studies did not control for confounding factors like arm length [26], which could interact with the drone position. Our human–drone proxemics study thus contributes to proxemic theory by mapping the vertical outlines of personal space. The bubble notion has to be replaced with a force field that is shaped quite differently when it comes to stimuli that are artifacts without the complexities introduced by human stimuli.

### 4.2. Effect of Technology and Real-World Characteristics

There were differences in the average comfortable interaction distance between the two experiments as well as in the shape of the height dependency for the simple spherical drone and the more complex drones. In Experiment 1, the average preferred distance across all heights and angles was 1.84 m and was therefore greater than the average preferred distance across all heights in Experiment 2 with 1.56 m. The type of AR technology differed between the experiments. Experiment 1 used a headset with optical see-through (OST), whereas Experiment 2 used video see-through (VST). A comparison of distance estimation with OST and VST has shown that subjects underestimated the distance to augmented objects with both technologies, but this underestimation was more pronounced with VST than with OST [27]. Although this does not entirely explain the observed differences between our experiments, it may be a contributing factor to consider.

Moreover, the characteristics of the rooms in which the two experiments were conducted could explain the differences we found between the two experiments for the comfortable interaction distance. In Experiment 1, the drone blended in very well with the plain room with its white walls and grey elements (see Figure 1). In contrast, in Experiment 2, the drone was more distinct from the real world due to the different colors and lighting conditions in the room (see Figure 5). This might also explain why the subjects preferred a larger distance to the human avatar; it was larger than expected based on previous research on human–human interaction with real-world humans and avatars in a fully immersive virtual environment [1,28]. Maybe there was something uncanny about inserting virtual humans into the real world [29].

Furthermore, this effect of uncanniness could have impacted the size estimation accuracy of the drone diameter. In Experiment 1 with OST and the plain room, the size was estimated quite accurately, whereas in Experiment 2, where the drone was more distinct from the real world, the size of the drone was significantly overestimated.

### 4.3. Drone Appearance

The drone’s appearance had a significant effect on the comfortable interaction distance. The mere suggestion of a face by adding simple eyes and ears caused subjects to perceive the drone model as more human. Surprisingly, this did not lead to a situation where the subjects wanted this drone model to come closer. Rather, it led to a desire for a greater distance at particularly low and high levels of height. Some of the subjects justified this by expressing the desire to have eye contact with the drone. The intermediate height also afforded manual interaction. Thus, the drone was likely perceived as a social actor and social norms from human–human interaction were activated, such as looking each other in the eyes during communication or reaching out for a hand-shake. The role of social norms in human–machine interaction has been the subject of research in diverse contexts, as outlined in the systematic literature review by Ribino [30] on politeness as a universal social norm, for example.

The additional presence of attachment to the ground by means of the simple feet led to a greater comfortable distance in nearly all cases compared to the drone models without attachment to the ground. It is unclear whether this was due to the ground anchoring itself and the associated option of tipping over, or whether this was due to the perceived unnaturalness in the room, as mentioned above, or if objects attached to the ground were generally perceived to be closer. It is nonetheless remarkable that at the lowest height of 10%, which is just above the ground and outside the subject’s reach, the ground-anchored drone was allowed to approach the subjects in closest proximity. Some subjects mentioned that the specific combination of anthropomorphic features and the small legs made the model appear particularly cute. It is conceivable that, in this case, this model was perceived in a similar way to a pet and that this resulted in a reduced preferred distance.

### 4.4. Limitations

One of the study limitations is that our sample consisted primarily of young students with a higher affinity for technology than the general population, which may limit the generalizability of the results. The aforementioned potential influence of the additional weight on the head due to wearing the headset may limit the transfer to real-world applicability. Also, the augmented drone posed no real physical threat, possibly reducing the need for safety. Future research should explore how these findings transfer to real-world interactions with actual drones to understand the application and limits of studying human–drone interaction with AR. This understanding could support user interaction studies with early-stage prototypes without the investments in terms of time and finance needed for creating functional, real prototypes.

### 4.5. Implications and Future Research

What implications for the design of human–drone interaction can we identify based on the outcome of the two studies? Depending on the intended purpose of the drone, there are different heights at which it can and should be operated. Accordingly, heights at or below eye level should be preferred if possible. The desired degree of anthropomorphization is also likely to depend on the purpose of the drone. For instance, drones that are supposed to serve as household assistants will presumably have different requirements in terms of their appearance and interaction than delivery drones.

It is important to understand the extent to which social norms are activated in interaction with users, but also which social norms should be taken into account by the drone to facilitate a positive interaction between humans and drones. Future research should, therefore, investigate how characteristics attributed to humans affect interaction with drones and the preferred distance to them. Among these characteristics are, for example, actual communication through eye contact, facial expression, or voice. In this regard, the recognition of human emotional states by drones constitutes an increasingly important aspect of research. A variety of approaches make use of sensors to obtain information about users, which is then interpreted using machine learning algorithms in order to draw conclusions about their emotional state (see, e.g., [31]). The interaction parameters, e.g., distance or approaching speed, can then be adapted accordingly.

This is particularly relevant when considering the finding that our distance requirement grows substantially when the drone position exceeds eye height, which allows for specific design recommendations. If close proximity is required, for example, when handover is necessary for the interaction and the drone approaches above eye level (see, e.g., [32]), great care should be taken to make its appearance from this perspective user-friendly. Designers should avoid features such as sharp edges or unprotected rotating parts, which could be perceived as causing injury in case of unforeseen drone movements.

In addition, further studies should examine the extent to which results obtained in AR can be transferred to real-life situations. This could enable future drone development processes to be more efficient and cost-effective.

## 5. Conclusions

We have conducted two experiments to investigate the factors that determine the preferred distance human observers would like to keep from a benign drone. We found a consistent height dependence of the preferred distance to the effect that the smallest distances are preferred at about 50% of one’s own eye level. Surprisingly, the anthropomorphic appearance of the drone produced larger distance preferences. This highlights the importance of further research to optimize human–drone interaction.

## Data Availability

Data are available in an OSF repository: https://osf.io/xjnr3/.
